# Using NMR-Based Metabolomics to Evaluate Postprandial Urinary Responses Following Consumption of Minimally Processed Wheat Bran or Wheat Aleurone by Men and Women

**DOI:** 10.3390/nu8020096

**Published:** 2016-02-17

**Authors:** Ramandeep Garg, Lorraine Brennan, Ruth K. Price, Julie M. W. Wallace, J. J. Strain, Mike J. Gibney, Peter R. Shewry, Jane L. Ward, Lalit Garg, Robert W. Welch

**Affiliations:** 1 The Northern Ireland Centre for Food and Health (NICHE), Ulster University, Coleraine BT52 1SA, UK; ramandeep.garg@um.edu.mt (R.G.); j.wallace@ulster.ac.uk (J.M.W.W.); jj.strain@ulster.ac.uk (J.J.S.); rw.welch@ulster.ac.uk (R.W.W.); 2 UCD Institute of Food and Health, University College Dublin, Belfield, Dublin 4 D04V1W8, Ireland; lorraine.brennan@ucd.ie (L.B.); mike.gibney@ucd.ie (M.J.G.); 3 Department of Plant Science, Rothamsted Research, Harpenden, Hertfordshire AL5 2JQ, UK; peter.shewry@rothamsted.ac.uk (P.R.S.); jane.ward@rothamsted.ac.uk (J.L.W.); 4 School of Agriculture, Policy and Development, University of Reading, Whiteknights, Reading, Berkshire RG6 6AH, UK; 5 Computer Information Systems, Faculty of Information and Communication Technology, University of Malta, Msida MSD 2080, Malta; lalit.garg@um.edu.mt

**Keywords:** NMR metabolomics, wheat bran, wheat aleurone, energy metabolism, sex differences, urine, lactate, alanine, *N*-acetylaspartate, *N*-acetylaspartylglutamate, betaine, hippurate

## Abstract

Wheat bran, and especially wheat aleurone fraction, are concentrated sources of a wide range of components which may contribute to the health benefits associated with higher consumption of whole-grain foods. This study used NMR metabolomics to evaluate urine samples from baseline at one and two hours postprandially, following the consumption of minimally processed bran, aleurone or control by 14 participants (7 Females; 7 Males) in a randomized crossover trial. The methodology discriminated between the urinary responses of control, and bran and aleurone, but not between the two fractions. Compared to control, consumption of aleurone or bran led to significantly and substantially higher urinary concentrations of lactate, alanine, *N*-acetylaspartate acid and *N*-acetylaspartylglutamate and significantly and substantially lower urinary betaine concentrations at one and two hours postprandially. There were sex related differences in urinary metabolite profiles with generally higher hippurate and citrate and lower betaine in females compared to males. Overall, this postprandial study suggests that acute consumption of bran or aleurone is associated with a number of physiological effects that may impact on energy metabolism and which are consistent with longer term human and animal metabolomic studies that used whole-grain wheat diets or wheat fractions.

## 1. Introduction

Epidemiological studies have demonstrated that higher consumption of whole-grain foods may reduce the risk of chronic diseases, such as cardiovascular diseases (CVD), diabetes, and some cancers [1]. Many hypotheses for the health-protective mechanisms of whole-grains have been proposed; however, the specific factors and the underlying mechanisms responsible for these health benefits remain unclear [2]. Wheat is a dietary staple in many regions, and whole-grain wheat contains many potentially bioactive components including dietary fiber, vitamins, minerals and phenolics such as ferulic acid, and the physiological methyl donors, betaine and choline [2]. These components are concentrated in the bran fraction which accounts for 15%–16% of the whole-grain and which is separated when wheat grain is milled to yield refined white flour [3]. In bran, these components are further concentrated in the metabolically active aleurone layer that comprises 45%–50% of the bran produced in milling and which can be isolated for use as a food ingredient that may confer beneficial physiological effects [2,3].

Indeed, a four-week intervention study in our laboratory found that consumption of aleurone-rich foods impacted favorably on a number of biomarkers of health, including decreasing plasma concentrations of total homocysteine (tHcy), LDL cholesterol and the inflammatory marker, C-reactive protein [4,5]. The decrease in plasma tHcy, which was accompanied by increased plasma betaine concentrations, was attributed to enhanced activity of betaine-homocysteine methyltransferase (BHMT) [4]. However, it was unclear if the potentially favourable changes in other biomarkers were attributable to one component or a combination of components, acting by one or more mechanisms [5].

Metabolomics, which is the complete analysis of low molecular weight metabolites is increasingly being used to evaluate the complex effects of diets on metabolism [6], and a number of metabolomic studies of varying design and duration, have used humans or animal models to evaluate the effects of diets containing various whole-grain foods or whole-grain fractions on plasma and/or urinary metabolite profiles. However, very variable results have been reported [7,8,9,10,11,12,13]. A recent comprehensive review that assessed the role of metabolomics in exploring the mechanisms underlying the health benefits of diets that are high in whole-grains concluded, inter alia, that heterogenous results are to be expected when there are wide variations in the studies such as the characteristics of the populations or participants, study duration, and the nature of the intervention products [14].

In a previous study, which used conventional analytical methods, we showed significant and substantial increases in plasma betaine and ferulic acid concentrations and in urinary ferulic acid excretion up to at least three hours postprandially following the consumption of relatively large amounts (50 g) of minimally processed wheat bran or aleurone [15,16]. In the present investigation, we have used NMR metabolomics to evaluate urine samples that were available from our previous study in order to give further information on postprandial responses.

## 2. Materials and Methods

### 2.1. Participants and Study Design

Full details of the study design, treatments and participants have been previously reported [15]. Briefly, 17 participants were initially recruited but three did not enter the study. Thus, there were 14 participants (seven males and seven females) recruited from the staff and students at Ulster University, Coleraine, UK. The participants met the following inclusion criteria: 18–40 years, BMI 18–30 kg/m^2^, non-smokers, without any clinical disorders, food allergies or intolerance, not taking any medication or supplements, and women not pregnant or lactating. Participants gave written informed consent prior to the commencement of the study and all data were anonymized. The study was approved by the Ulster University Research Ethics Committee and was registered on the Current Controlled Trials register (ISRCTN09560399). The study consisted of three morning sessions, at least one week apart, and was a randomized crossover design with at least 1–week washout between each test day. The treatments were (1) minimally processed wheat bran (50 g); (2) minimally processed wheat aleurone (50 g) and (3) a balanced control. On the two days prior to each occasion, participants adhered to a low phenolic diet which restricted their intake of foods with high phenolic content, including whole grains, fruit, coffee, tea, *etc.* [15]. On the morning of the test day, participants were instructed to empty their bladders and drink 250 mL of uncarbonated water around one hour before attending the Ulster University. On arrival at the University, between 08.00 and 09.00 h, having fasted from 22.00 h the previous night, baseline urine samples were collected and treatments served for consumption over the next 15 min with 500 mL of uncarbonated water. Further urine samples were collected at one, two, three and four hours postprandially; however, due to resource constraints, in the present study, urine samples were analyzed only from baseline and one and two hours. Urine samples collected were aliquoted and stored frozen at −80 °C until ^1^H NMR analysis (University College Dublin, Ireland).

### 2.2. Preparation and Analysis of the Treatments

The sources of ingredients, the method of preparation and the treatments were previously reported in detail [15]. Briefly, the treatments were formulated to balance macronutrient and fiber contents using refined ingredients and analyzed for betaine [17] and phenolic acids [18] (Rothamsted Research). The ingredients, formulations and calculated nutrient and energy composition of treatments are given in Table 1.

### 2.3. NMR Spectroscopy

Urine samples were prepared by the addition of 200 μL phosphate buffer (0.2 mol/L KH2PO4, 0.8 mol/L K_2_HPO_4_) to 500 µL urine. Following centrifugation at 8000× *g* for 5 min, 10 μL sodium trimethylsilyl (2,2,3,3–^2^H_4_) propionate (TSP) and 50 μL deuterium oxide (D_2_O) were added to 550 μL of the supernatant. Sodium trimethylsilyl propionate (TSP) was used as a chemical shift reference and 10% D_2_O as a lock solvent for high resolution NMR spectrum. A 500 MHz DRX NMR spectrometer (Bruker Biospin, Karlsruhe, Germany) was used to acquire spectra with 8 kHz spectral width, 128 scans into 32 K data points, with 2.5 s relaxation delay between successive scans. Using a Noesypresat pulse sequence, solvent suppression of residual water signal was achieved during the relaxation delay and the mixing time of 100 ms. Spectra alignment was achieved using SpecAlign [19].

### 2.4. NMR Spectra Pre-Processing

NMR spectra were first processed using Bruker software with a line broadening of 0.2 Hz and each spectrum was manually baseline corrected. The spectra were integrated into 0.04 ppm regions excluding the water region (4–6 ppm) using AMIX software (Bruker Biospin, Karlsruhe, Germany). The spectral intensities were normalized to the total spectral intensity ensuring the uniform strength of all samples by removing the variability among them.

### 2.5. Data Analysis

Multivariate analysis of ^1^H NMR data was carried out using SIMCA-P+ (version 11.5.0.0; Umetrics AB, Umeå, Sweden). The spectral data were imported into SIMCA and pareto scaled. Unsupervised principal component analysis (PCA) was applied to the data for initial visualization, inspection of trends, identification of outlying data (outside the 95% confidence region based on Hotelling T^2^ of the model). To explore further any trends in the data, partial least square discriminant analysis (PLS-DA) was employed. The quality of PLS-DA models was evaluated using *R*^2^, an estimate of goodness of fit of model to the data, and *Q*^2^, an estimate of goodness of prediction [20]. In addition, a cross validation step was performed where two thirds of the observations were randomly selected to form a training set to train the model which was then used to determine the class memberships of the test set containing the remaining third of the observations. This process was repeated three times such that each observation was predicted exactly once. For each model, the predictive ability was calculated as the average percentage of observations classified correctly. Discriminating bin regions were identified by examination of the loadings plot and the VIP (variable importance in projection) values. Variables having VIP larger than 1 were considered most important [21]. Metabolites were identified by use of in-house libraries and the Chenomx library.

## 3. Results

### 3.1. Composition of the Treatments

The energy, macronutrient and fiber contents of the aleurone, bran and control treatments were similar (Table 1). However, compared to the control, the bran was very much higher in betaine, total phenolic acids and total ferulic acid content, and the aleurone had the highest amounts of these components (Table 1).

### 3.2. Participant Characteristics and Compliance

Fourteen participants (7 males, 7 females; 27.8 ± 6.5 years; BMI 22.7 ± 2.6 kg/m^2^) completed the study and no significant difference in compliance (*p* = 0.207) was found. Overall treatments compliance (%; mean ± SD) was 96 ± 9.7, while compliance was 96.1 ± 6.5 (range 83–100), 93.4 ± 15.4 (range 48.1–100) and 100 ± 0, for the aleurone, bran and control treatments, respectively.

### 3.3. Metabolomic Analysis of Urine Samples

**Figure 1:**
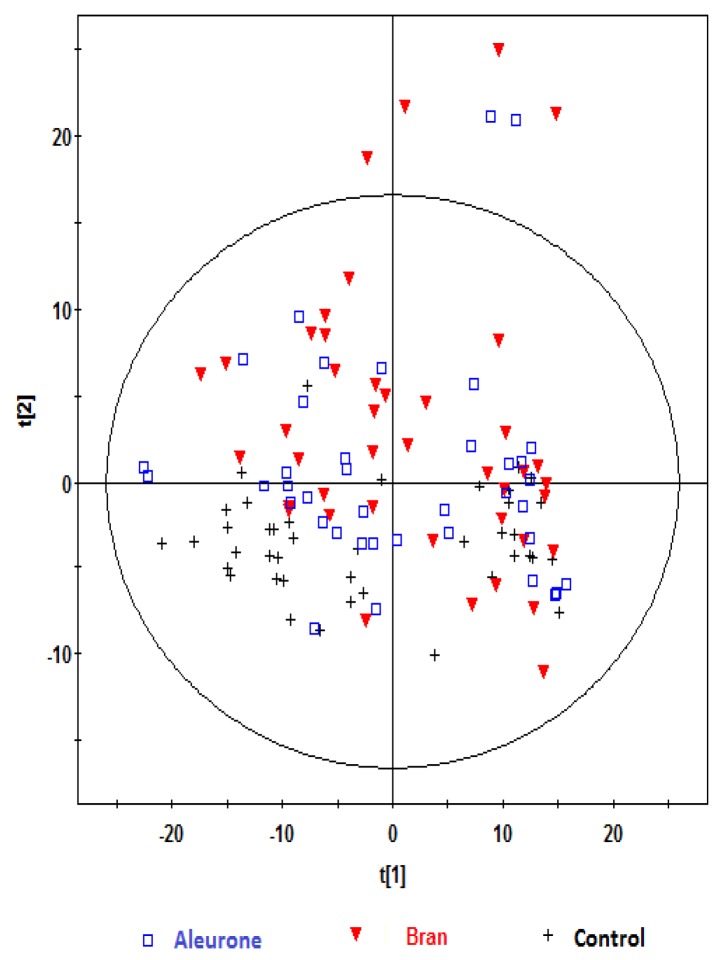
Principal component analysis (PCA) scores plot t[1] *vs.* t[2] obtained from ^1^H NMR spectra of urine samples of fourteen participants at baseline and at one and two hours after consumption of aleurone, bran or control treatments, showing the different treatments. Observations (*n*) = 122. The ellipse represents 95% confidence region of the model based on Hotelling T^2^.

**Figure 2:**
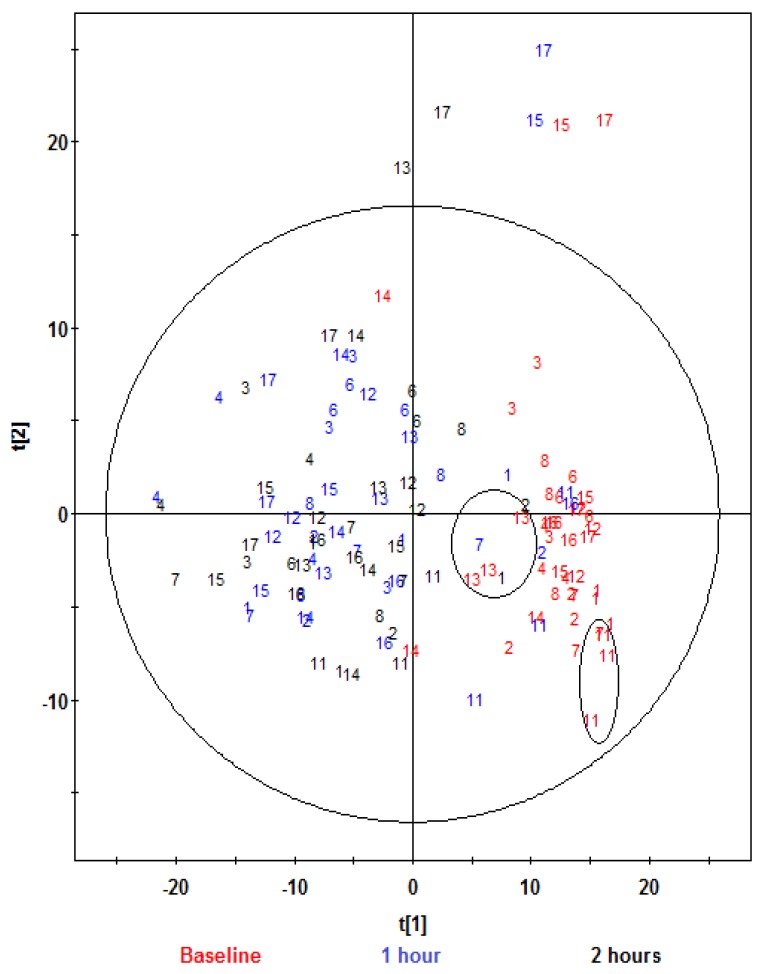
PCA scores plot t[1] *vs.* t[2] obtained from ^1^H NMR spectra of urine samples of fourteen participants at baseline and at one and two hours after consumption of aleurone, bran or control treatments, showing the different time-points and participant identifying numbers. Observations (*n*) = 122 (17 participants were recruited, but participants 5, 9 and 10 did not enter the study. Samples from four participants gave poor quality spectra and were excluded (participant 2, aleurone, 2 h; participant 3, aleurone, 2 h; participant 4, control, 2 h; participant 8, bran, 1 h). The ellipse represents 95% confidence region of the model based on Hotelling T^2^.

The PCA scores plots of ^1^H NMR data in Figure 1 give an overview of the profiles for the respective treatments. Figure 2 shows the same PCA scores as in Figure 1, but with their corresponding time-points of sample collection. Six observations were identified as outliers as these were lying outside the 95% confidence region of the model based on Hotelling T^2^ and excluded before further analyses (Figure 1 and Figure 2). Visual inspection of Figure 1 indicated that the control samples were located mainly in the lower two quadrants and were differentiated from the other treatments. Visual inspection of the PCA score plot in Figure 2 showed that the baseline samples were grouped in the right quadrants, and differentiated from the one and two hours postprandial samples, which were mainly grouped in the left quadrants, and not differentiated from each other. Furthermore, observation of the baseline samples (Figure 2) suggests that the intra-participant variation (variation among the baseline samples collected for the same participant on different dates) was relatively low, and was considerably less than inter-participant variation (variation between different participants considering only baseline samples). See, for example, baseline samples for participants 11 and 13, which are circled in Figure 2.

**Figure 3:**
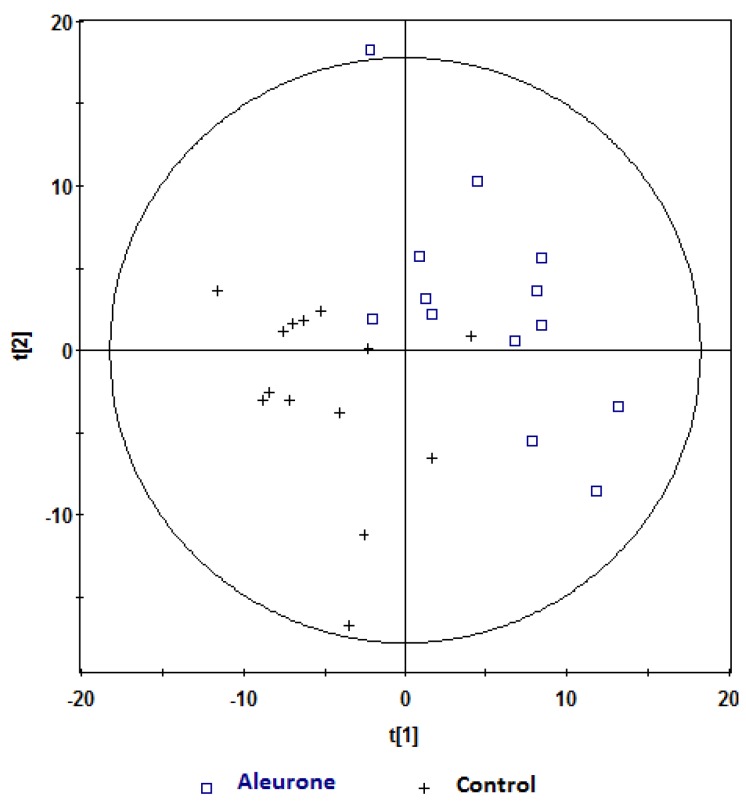
PLS-DA scores plot (t[1]) *vs.* t[2]) obtained from ^1^H NMR spectra of postprandial urine samples of aleurone *vs.* control at one hour (Observations, *n* = 27), 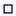, aleurone; 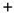, control. Variables (K) = 181. PLS component 1: *R*^2^ = 0.196, *Q*^2^ = 0.315; PLS component 2: *R*^2^ = 0.18, *Q*^2^ = 0.117. The ellipse represents 95% confidence region of the model based on Hotelling T^2^.

**Figure 4:**
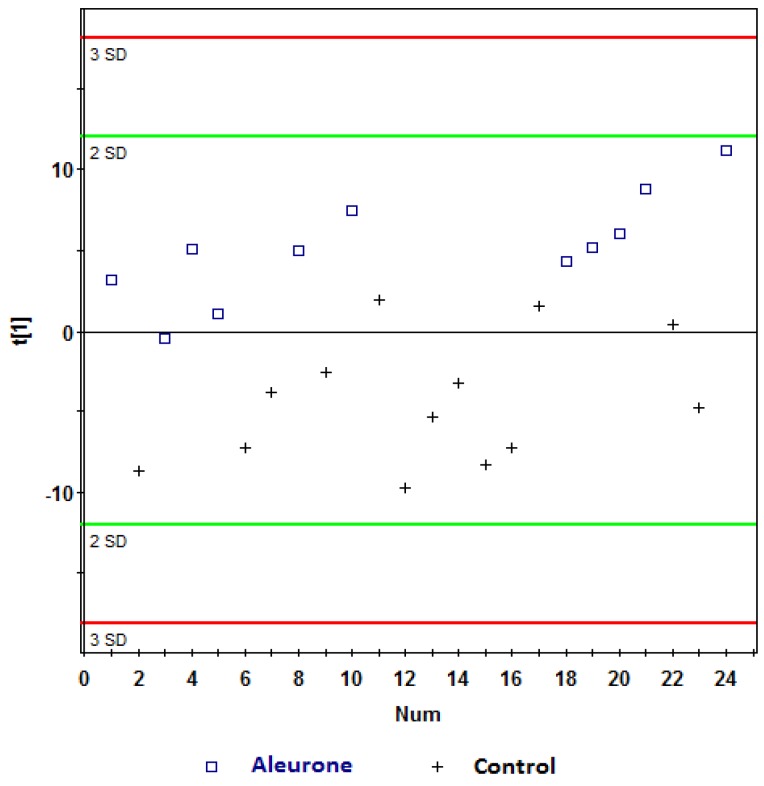
PLS-DA scores plot (t[1]) *vs.* t[2]) obtained from ^1^H NMR spectra of postprandial urine samples of aleurone *vs.* control at two hours (Observations (*n*) = 24), 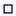, aleurone; 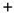, control. Variables (K) = 181. PLS component 1: *R*^2^ = 0.148, *Q*^2^ = 0.339.

**Figure 5:**
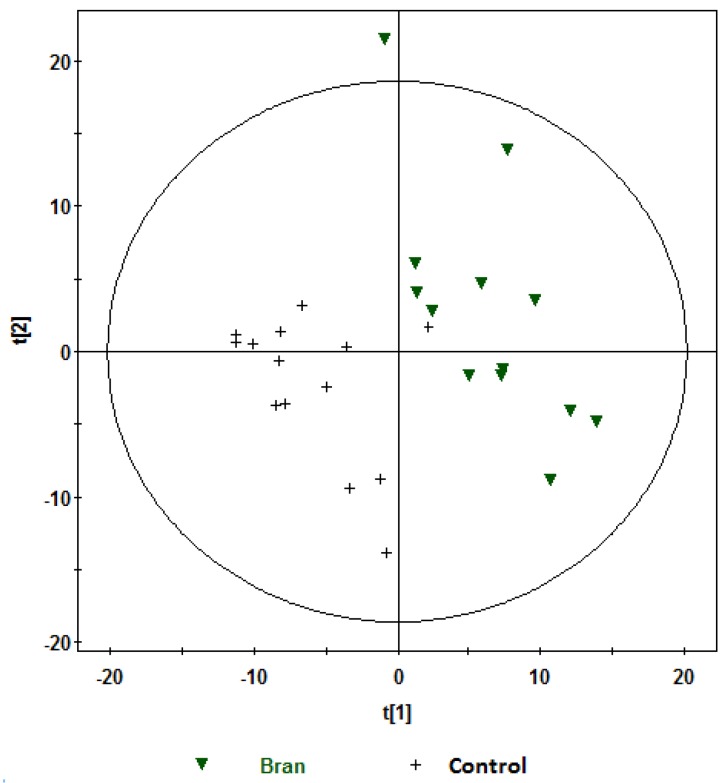
PLS-DA scores plot (t[1]) *vs.* t[2]) obtained from ^1^H NMR spectra of postprandial urine samples of bran *vs.* control at one hour (Observations (*n*) = 27), 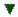, bran; 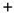, control. Variables (*K*) = 181. PLS component 1: *R*^2^ = 0.204, *Q*^2^ = 0.51; PLS component 2: *R*^2^ = 0.176, *Q*^2^ = 0.111. The ellipse represents 95% confidence region of the model based on Hotelling T^2^.

**Figure 6:**
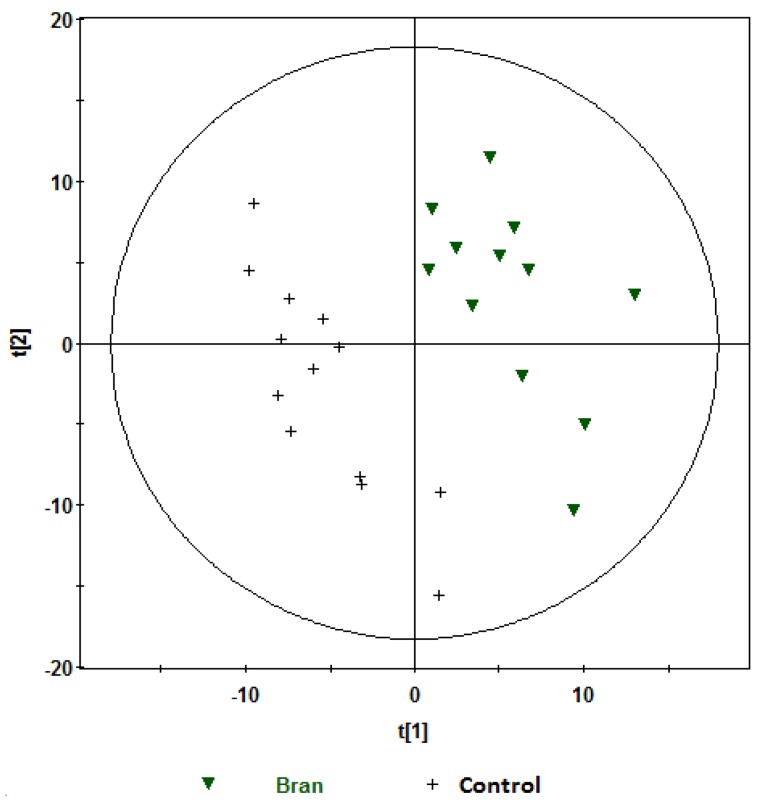
PLS-DA scores plot (t[1]) *vs.* t[2]) obtained from ^1^H NMR spectra of postprandial urine samples of bran *vs.* control at two hours (Observations (N) = 25). 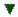, bran; 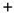, control. Variables (*K*) = 181. PLS component 1: *R*^2^ = 0.179, *Q*^2^ = 0.379; PLS component 2: *R*^2^ = 0.168, *Q*^2^ = 0.327. The ellipse represents 95% confidence region of the model based on Hotelling T^2^.

Further analyses were performed on the ^1^H NMR data using PLS-DA analysis. Robust models were built comparing aleurone *vs.* control and bran *vs.* control at one hour and two hours postprandially, and are shown in Table 2. These models had very good *Q*^2^ predictability, and comparable *R*^2^ goodness of fit. Further, the predictive ability of these models was cross-validated (Table 2). Visual inspection of PLS-DA score plots for the models showed clear separations between aleurone and control (Figure 3 and Figure 4) and bran and control (Figure 5 and Figure 6) at both one hour and two hours. However, when the aleurone and bran were compared, there were no PLS-DA models at either time-point.

### 3.4. Metabolite Identification

For each of the models discussed above, the VIPs, mean concentrations and differences in concentrations obtained from ^1^H NMR spectra were used to identify changes in metabolite profiles at the different time-points. These data are shown for all chemical shifts with VIP values greater than 1 in the Supplementary Materials in Tables S1–S4, for the aleurone *vs.* control at one and two hours, and the bran *vs.* control at one and two hours, respectively. Further analyses of PLS-DA models were carried out to identify the metabolites responsible for the variation explained by these models, and these discriminating metabolites and the relative changes in their concentrations are shown in Table 3. At one hour and two hours postprandial, urinary concentrations of lactate, alanine, *N*-acetylaspartate (NAA) and *N*-acetylaspartylglutamate (NAAG) were significantly and substantially higher, and betaine concentrations were significantly and substantially lower after consumption of the aleurone and bran compared to those after consumption of the control. Concentrations of 3-hydroxy-isovalerate and two unknown metabolites (at 3.68 and 3.88 ppm) were higher at one hour and two hours only after consumption of the bran relative to control. Other changes were less systematic; for those metabolites that could be identified, compared to the control, the concentration of citrate was higher only at one hour after consumption of aleurone, the concentration of hippurate was higher only at two hours after consumption of the aleurone, and the concentration of 2-hydroxyisobutyrate was higher only at two hours after consumption of bran (Table 3).

### 3.5. Comparisons between Sexes for Effects of Treatments

Comparisons of females and males for each treatment at each time-point showed significant sex-related differences at baseline and at one and two hours postprandially after the consumption of control and aleurone, but only at two hours after the consumption of bran (Table 4). Overall, in females compared to males, concentrations of the discriminating metabolites citrate and hippurate were generally higher, and concentrations of betaine were generally lower (Table 4).

## 4. Discussion

The results indicate that the methodology employed successfully discriminated between the urinary metabolite profiles at one and two hours after consumption of both aleurone and bran compared to the control (Table 2), but there was no clear discrimination between the aleurone and bran. As all treatments were balanced for macronutrients and fiber contents (Table 1), the findings indicate that the discrimination resulted from differences in the minor components present in the aleurone and bran, such as betaine or phenolics. Furthermore, identification of the discriminating metabolites underlying these differences showed that, compared to the control, consumption of the aleurone or bran led to significantly and substantially higher urinary concentrations of lactate, alanine, NAA and NAAG ,and significantly and substantially lower urinary betaine concentrations at both one and two hours postprandially (Table 3).

The mechanisms underlying the effects on urinary lactate and alanine are unclear. However, the cellular production of lactate and alanine from glucose and glutamine is associated with the Warburg effect [22]. Although interest in the Warburg effect has focused on tumor cells which rely on this pathway to generate high energy intermediates [22], this effect is also found in proliferating non-tumor cells [22,23]. Most of the lactate and alanine produced in this way is excreted by the cell as a waste product and, although they may be recycled, lactate and alanine are excreted in the urine [22]. Thus, we suggest that the higher urinary lactate and alanine observed here, one and two hours after the consumption of wheat bran or wheat aleurone compared to the control, reflects a perturbation in energy metabolism. Furthermore, although differences in study design make rigorous comparisons difficult, this suggestion is compatible with results from other studies that have evaluated urinary metabolic responses to the consumption of whole-grain wheat diets or wheat fractions, as outlined below.

Metabolomic analysis of the urine of male rats fed for two weeks on diets containing 60% whole-grain wheat flour or 60% refined wheat flour using a cross-over design showed that urinary excretion of some tricarboxylic acid cycle intermediates was significantly higher in rats fed the whole-grain diet [8]. Furthermore, comparison of the postprandial and post-absorptive urinary profiles showed significantly higher lactate, citrate and 2-oxoglutarate in the postprandial period [8]. A four-week cross-over trial with 11 women and six men compared diets rich in whole-grains or rich in refined grains [13]. Metabolomic analysis of urine samples collected after one and two weeks on each diet showed that, after one week, compared to the refined grain diet, the whole-grain diet led to lower urinary excretion of metabolites related to protein catabolism, lipid metabolism, gut microbial metabolism and central energy metabolism, but only in men [13]. However, there were no differences between the diets after two weeks intervention. The authors concluded that changes in a number of aspects of metabolism, including central energy metabolism, may provide mechanisms of action for the benefits of whole-grain diets [13]. Similar results were found in a study with female rats that were fed for 30 days on purified diets containing 15% wheat bran fiber or a low fiber control [9] and where the wheat bran diet was differentiated from the low fiber control by a number metabolites associated with protein metabolism, lipid metabolism, gut microbial metabolism and energy metabolism [9]. Furthermore, specific effects included significantly higher urinary lactate and alanine in the rats fed the 15% wheat bran fiber diet compared to the control [9] and the authors concluded that wheat bran fiber can affect energy metabolism in rats [9].

As has been previously reported, it was not possible to resolve NAA/NAAG in the NMR spectra [24]. However, there were consistent increases in urinary NAA/NAAG following consumption of the aleurone and bran compared to the control and, as far as we are aware, this is the first report that NAA/NAAG can be influenced by dietary factors. NAA and its derivative NAAG are found at high concentrations in the human brain [25], where the concentration of NAA is about tenfold that of NAAG [24], the most abundant neuropeptide in the brain [26]. NAA is found in human urine in low micromolar concentrations, reflecting the daily excretion of about 1% of the NAA in the brain [25]. Proposed roles for NAA include acting as a precursor for NAAG, acting as an osmolyte and facilitating energy metabolism in neuronal mitochondria [25]. NAA is synthesized solely in the mitochondria and its synthesis is coupled with mitochondrial energy production [25]. Thus, NAA decreases when brain mitochondrial energy production decreases [25] and furthermore, the close correlation between NAA synthesis and mitochondrial energy metabolism may reflect mitochondrial integrity and “well-being” [27]. Brain NAA concentrations are decreased in a number of disease states including ischemic stroke and brain tumors [25]. Furthermore, experimental impairments of brain energy metabolism lead to decreases in NAA concentrations [25], and decreases in NAA concentration are associated with mitochondrial dysfunction [24]. Thus, we suggest that the increases in urinary NAA/NAAG observed here may be associated with enhanced mitochondrial energy production in the brain.

Evidence from animal studies and other sources indicates that betaine can play important roles in energy metabolism and that betaine insufficiency is associated with, mitochondrial dysfunction [28]. Furthermore, a recent *in vitro* study with mouse hepatocytes has shown that exogenous betaine stimulated mitochondrial and cellular respiration within 30 min and highlighted the potential role of betaine in mitochondrial function and energy metabolism [29]. Thus, the previously reported substantial increases in plasma betaine concentrations that were apparent within 30 min of consumption of bran and aleurone and which persisted for at least three hours [15], suggest that the putative effects on energy metabolism observed here may be mediated, at least in part, by betaine.

It may appear somewhat surprising that, despite the previously reported increases in plasma betaine [15], consumption of aleurone and bran led to lower urinary betaine concentrations at both one and two hours postprandially, compared to the control (Table 3). However, urinary excretion of betaine is minimal and not correlated with plasma concentrations and, when betaine supplements are taken chronically, there are at most, small, transient increases in urinary betaine excretion [30]. Nevertheless, as previously described, plasma betaine concentrations peaked at 1–2 h postprandially and were 1.1 and 1.8 times greater than the baseline concentrations for the bran and aleurone respectively [15], whereas the urinary betaine concentrations decreased by a mean of 27% for the bran and 45% for the aleurone in the two hours postprandial (Table 3). These findings indicate that there may be a systematic negative association between plasma betaine concentrations and urinary betaine concentrations in this postprandial study. The mechanism(s) underlying such a putative association are unclear. However, betaine is metabolized to dimethylglycine (DMG) by betaine-homocysteine methyltransferase (BHMT) [30] and, as previously reported, the postprandial increases in plasma betaine concentrations were accompanied by increases in plasma DMG concentrations [15]. BHMT activity is influenced by a number of complex factors [30] and has been shown to increase in rats fed betaine or given betaine by intraperitoneal injection [31]. Thus, it is possible that the acute increases in plasma betaine concentrations following the consumption of bran or aleurone resulted in increased BHMT activity which not only metabolized the recently absorbed betaine, but also endogenous betaine, and thus lowering its urinary excretion.

Hippurate, which is a normal component of urine, is synthesized in the mitochondria from glycine and benzoic acid [32]. Benzoic acid can be derived from dietary phenolic compounds such as ferulic acid, the major phenolic acid in wheat and wheat aleurone, as a result of microbial action in the large intestine [32]. However, previous metabolomic studies with wheat products have given contrasting results. A study with humans did not report any effects on urinary hippurate [13], whereas two studies with rats reported, contrastingly, higher [8] and lower [9] urinary hippurate concentrations. Our results showed higher urinary hippurate, but only at two hours after consumption of the aleurone. This relatively rapid response suggests that microbial activity may not be essential for the conversion of phenolics such as ferulic acid into the benzoate precursor of hippurate.

We found sex related differences in urinary metabolite profiles, with higher hippurate and citrate, and lower betaine in females compared to males. Higher urinary hippurate in females compared to males has also been found in metabolomic studies with rats and mice [33,34] and in human population studies [35,36]. Furthermore, higher urinary citrate in women compared to men has also been found in a longer term metabolomic study investigating the effects of chamomile consumption [37]. The current study appears to be the first report of sex differences in urinary betaine. Furthermore, these differences are in line with previous reports that have found lower fasting plasma betaine in women compared to men [15,30].

Participants were instructed to adhere to a low-phenolic diet on the two days prior to each meal, and during these two days the participants were provided with all meals and snacks. Standardizing diets can help reduce the variation in urinary metabolite profiles [38,39,40,41]. Consequently, the samples taken at baseline on each meal occasion followed two days of dietary standardization, and it was found that intra-participant variation tended to be lower in these samples than in the postprandial samples. These findings are consistent with the previous work [40], which showed comparatively low intra-participant variation and higher inter-participant variation in fasting urinary metabolite profiles after standardizing diets for one day, compared to non-standardized diets.

This exploratory study has a number of potential limitations which include the possibility of over fitting the data when using PLS-DA models, and the low number of participants and their relatively narrow age range and BMI status. Furthermore, the present study used large amounts (50 g) of bran and aleurone in order to maximize postprandial responses and to provide insights into potential mechanisms of action. Bran and aleurone are available as ingredients; however, to attain an intake of 50 g bran or 50 g aleurone from whole-grain wheat would require consumption of about 320 g and 680 g whole-grain wheat respectively. Thus, these levels are very much higher than the whole-grain intake of 151 g per day achieved in the previous whole-grain human intervention that evaluated metabolic responses [13].

Overall, the present results showed that the methodology could discriminate between the postprandial urinary metabolite profiles following consumption of control compared to the aleurone or bran, but it could not discriminate between the aleurone and bran. However, the changes in metabolites indicated postprandial perturbations in energy metabolism that are consistent with the results of longer term animal and human metabolomic studies that used wholegrain wheat diets or wheat fractions and which demonstrated, inter alia, changes in energy metabolism [8,9,13].

## Figures and Tables

**Table 1:** Formulation and composition of the treatments.

	Aleurone	Bran	Control
Ingredients (g per portion) ^a^			
Wheat bran ^b^	-	50.0	-
Wheat aleurone ^b^	50.0	-	-
Wheat fiber ^c^	3.6	-	27.0
Wheat protein ^d^	-	2.3	8.9
Wheat starch ^e^	-	0.3	5.6
Vegetable fat ^f^	-	0.6	2.0
Sugar ^g^	2.5	2.5	2.5
Composition per portion			
Energy (kJ)	352	348	336
Carbohydrate (g)	8.1	8.0	7.4
Protein (g)	8.9	8.7	8.4
Fat (g)	2.0	2.0	2.1
Fiber (g)	27	27	25
Betaine (mg)	515	301	31
Total phenolic acids (mg) ^h^	213	162	1.5
Total ferulic acid (mg) ^i^	177	124	0.9
Free ferulic acid (mg)	2.2	1.2	0.0

^a^ Fresh weight basis; ^b^ Supplied by Bühler AG Uzwil, Switzerland; ^c^ Vitacel^®^ 600, J. Rettemmaier & Söhne GmbH, Rosenberg, Germany; ^d^ Gluten AG110, Syral, Aalst, Belgium; ^e^ Meritena 200, Syral, Aalst, Belgium; ^f^ Cookeen, Princes Ltd, Liverpool, UK; ^g^ White granulated sugar, local store; ^h^ Suml of ferulic acid, 4-hydroxybenzoic acid, vanillic acid, syringic acid, syringaldehyde, caffeic acid, 2,4-dihydroxybenzoic acid, sinapic acid and *p*-coumaric acid; ^i^ Sum of free, conjugated and bound ferulic acid.

**Table 2:** Summary of parameters for assessing PLS-discriminant analysis (DA) models.

Treatments	Time-Point (h)	No. of Components	^a^ *R*^2^	^b^ *Q*^2^	Cross Validation (%) ^c^
Aleurone *vs.* control	1	2	0.377	0.395	74
Aleurone *vs.* control	2	1	0.148	0.339	71
Bran *vs.* control	1	2	0.380	0.564	93
Bran *vs.* control	2	2	0.347	0.582	93

^a^ multiple correlation coefficient (*R*^2^) an estimate of goodness of fit of model to the data; ^b^ cross validation correlation coefficient (*Q*^2^) an estimate of goodness of prediction; **^c^** average % of samples correctly classified during cross validation.

**Table 3:** Discriminating metabolites (with respect to control) at one hour and two hours postprandial following aleurone and bran.

Chemical Shift (ppm) ^a^	Discriminating Metabolites	Percentage (%) Change in Metabolite Concentration with Respect to Control			
		Aleurone (1 h)	Aleurone (2 h)	Bran (1 h)	Bran (2 h)
1.32	Lactate	97	109	94	187
3.24	Betaine	−47	−43	−27	−27
1.52	Alanine	43	40	52	61
2.16, 2.20	NAA/NAAG ^b^	65	59	78	110
2.52, 2.56, 2.64, 2.68	Citrate	24	-	-	-
7.56, 7.84	Hippurate	-	35	-	-
3.48, 3.52, 3.60	Unknown	-	-	66	-
1.28	3-hydroxyisovalerate	-	-	69	70
1.36	2-hydroxyisobutyrate	-	-	-	27
3.68	Unknown	-	-	18	34
3.88	Unknown	-	-	25	41

^a^ Chemical shifts having a variable importance in projection (VIP) value greater than 1 with 95% confidence (Jack-knifing (JK)) were considered as most important; ^b^ NAA: *N*-acetylaspartate; NAAG: *N*-acetylaspartylglutamate.

**Table 4:** Comparison between the sexes for urinary metabolite profiles after different treatments at different time-points.

Treatment	Timepoint (h)	Females (F) *vs* Males (M)	No. of Components	^a^ *R*^2^	^b^ *Q*^2^	Cross Validation (%) ^c^	Discriminating Metabolites ^d^
None	0	F *vs.* M	2	0.236	0.721	95	In females: citrate↑, hippurate↑, betaine↓
Aleurone	1	F *vs.* M	3	0.567	0.903	92	In females: citrate↑, hippurate↑, betaine↓
Aleurone	2	F *vs.* M	2	0.454	0.717	72	In females: citrate↑, hippurate↑
Bran	1	F *vs.* M	0	-	-	-	-
Bran	2	F *vs.* M	1	0.194	0.287	Not validated	In females: citrate↑, lactate↑
Control	1	F *vs.* M	2	0.372	0.304	61	In females: hippurate↑, betaine↓
Control	2	F *vs.* M	2	0.484	0.587	83	In females: hippurate↑, citrate↑, betaine↓

^a^ multiple correlation coefficient (*R*^2^) an estimate of goodness of fit of model to the data; ^b^ cross validation correlation coefficient (*Q*^2^) an estimate of goodness of prediction; ^c^ average % of samples correctly classified during cross validation; ^d^ only metabolites with significantly different concentrations, ↑ significantly higher in females, ↓ significantly lower in females than males.

## Supplementary Materials

The following are available online at http://www.mdpi.com/2072-6643/8/2/96/s1, Table S1: VIPs, mean concentrations and differences in concentrations from ^1^H NMR spectra of postprandial urine samples of aleurone *vs.* control at one hour for all chemical shifts having VIP value (for the first component) greater than 1 with 95% confidence (Jack-knifing (JK), Table S2: VIPs, mean concentrations and differences in concentrations from ^1^H NMR spectra of postprandial urine samples of aleurone *vs.* control at two hours for all chemical shifts having VIP value (for the first component) greater than 1 with 95% confidence (Jack-knifing (JK), Table S3: VIPs, mean concentrations and differences in concentrations from ^1^H NMR spectra of postprandial urine samples of bran *vs.* control at one hour for all chemical shifts having VIP value (for the first component) greater than 1 with 95% confidence (Jack-knifing (JK), Table S4, VIPs, mean concentrations and differences in concentrations from ^1^H NMR spectra of postprandial urine samples of bran *vs.* control at two hours for all chemical shifts having VIP value (for the first component) greater than 1 with 95% confidence (Jack-knifing (JK).
