# Supplementary material for: Using NMR-Based Metabolomics to Evaluate Postprandial Urinary Responses Following Consumption of Minimally Processed Wheat Bran or Wheat Aleurone by Men and Women
Source: Nutrients. 2016 Feb 17;8(2):96. doi: 10.3390/nu8020096 (PMC4772058; doi:10.3390/nu8020096)
Supplement: Supplementary file 1 [file nutrients-08-00096-s001.pdf]

Supplementary Materials: Using NMR-Based Metabolomics to Evaluate Postprandial Urinary Responses Following Consumption of Minimally Processed Wheat Bran or Wheat Aleurone by Men and Women

Ramandeep Garg, Lorraine Brennan, Ruth K. Price, Julie M. W. Wallace, J. J. Strain, Mike J. Gibney, Peter R. Shewry, Jane L. Ward, Lalit Garg and Robert W. Welch

Table S1. VIPs, mean concentrations and differences in concentrations from ^{1}H NMR spectra of postprandial urine samples of aleurone *vs.* control at one hour for all chemical shifts having VIP value (for the first component) greater than 1 with 95% confidence (Jack-knifing (JK).

Chemical Shift (ppm)	VIP Component 1	Aleurone		Control		Difference	%Difference
		Mean	S D	Mean	S D		
1.32	4.16249	14.27098	3.062182	7.251501	3.532878	−7.0194747	96.8
3.24	4.02001	8.238791	2.588537	15.48616	5.210814	7.24736637	−46.8
1.56	2.76565	6.473607	1.496637	3.359924	1.474532	−3.1136826	92.7
1.28	2.64036	8.245262	2.292278	5.049026	1.458486	−3.1962359	63.3
2.20	2.57021	6.966328	1.482933	4.224484	1.237119	−2.7418449	64.9
3.04	2.52177	131.2192	21.17296	140.6509	25.05072	9.43164082	−6.7
2.16	2.40019	9.034263	2.01052	6.329951	1.294214	−2.7043116	42.7
3.68	2.04940	24.73493	2.915893	21.96961	2.613812	−2.765314	12.6
3.56	2.04011	21.48823	12.23908	16.69236	4.407347	−4.7958611	28.7
2.64	1.91181	17.03774	4.481938	13.79421	4.72138	−3.2435321	23.5
3.20	1.82369	10.11876	2.830314	12.41903	2.017475	2.30026418	−18.5
2.56	1.82318	17.86715	4.32289	14.81978	4.639181	−3.0473721	20.6
0.88	1.80630	6.056719	1.614553	4.274602	1.145499	−1.7821171	41.7
2.68	1.77217	11.47222	2.989625	9.088912	2.670513	−2.3833079	26.2
7.12	1.72949	7.563787	2.595824	10.1234	4.151092	2.55960879	−25.3
7.56	1.67562	8.993662	5.203298	6.364859	2.274758	−2.6288023	41.3
2.52	1.60445	12.81225	2.705371	10.60473	3.280017	−2.2075179	20.8
1.36	1.44145	6.628033	1.925712	5.156502	1.243227	−1.4715309	28.5
1.48	1.42126	7.383495	2.577708	5.792986	1.149702	−1.5905089	27.5
1.52	1.38900	4.552364	1.597154	3.174042	1.449549	−1.3783217	43.4
3.72	1.32316	24.77209	6.872864	27.50995	7.370964	2.73785758	−9.9
8.20	1.27111	1.62975	0.745981	2.541526	0.74379	0.91177571	−35.9
6.20	1.23316	2.059606	1.095524	3.157927	1.341406	1.09832099	−34.8
3.88	1.21410	12.07729	3.242255	10.51419	2.078541	−1.5631039	14.9
3.28	1.20412	39.61967	14.88219	36.19298	12.36631	−3.4266905	9.5
7.84	1.18946	9.148038	5.364928	7.251094	2.732067	−1.8969441	26.2
6.04	1.15493	2.670983	1.364873	3.718709	1.2259	1.04772621	−28.2
3.36	1.14254	5.396922	0.836671	4.497122	1.047115	−0.8997994	20.0
1.40	1.10427	3.597015	1.390195	2.679131	0.671401	−0.9178847	34.3
0.80	1.09112	1.663855	0.736458	2.486962	0.98416	0.82310676	−33.1
8.12	1.08660	1.564265	0.772817	2.418736	1.103402	0.85447033	−35.3
8.16	1.05923	1.594912	0.741849	2.304637	0.604794	0.70972484	−30.8
8.08	1.03982	1.746189	0.660921	2.435821	0.663232	0.68963148	−28.3
7.88	1.02049	1.514524	0.793137	2.213375	0.634138	0.69885115	−31.6
6.40	1.01839	1.843823	0.809777	2.560051	0.70907	0.71622764	−27.9
8.24	1.00073	1.807235	0.983574	2.607875	1.033628	0.80064038	−30.7

Table S2. VIPs, mean concentrations and differences in concentrations from ^{1}H NMR spectra of postprandial urine samples of aleurone *vs.* control at two hours for all chemical shifts having VIP value (for the first component) greater than 1 with 95% confidence (Jack-knifing (JK).

Chemical Shift (ppm)	VIP Component	Aleurone		Control		Difference	%Difference
		Mean	S D	Mean	S D		
1.32	4.12820	10.58116	2.690213	5.068215	1.417053	−5.51294	108.8
3.24	3.99567	8.889558	1.857856	15.48453	5.350281	6.594973	−42.6
3.92	3.25484	17.91443	7.870071	11.68593	4.777831	−6.2285	53.3
3.68	2.96992	26.68105	3.804554	21.92234	4.805783	−4.75872	21.7
3.04	2.76697	130.0666	32.05445	140.5462	22.52962	10.47969	−7.5
2.16	2.56255	8.651467	2.097343	5.860334	1.571695	−2.79113	47.6
1.56	2.55878	6.246367	1.034049	3.741607	1.60901	−2.50476	66.9
2.20	2.52924	6.39849	1.257234	4.019585	1.22371	−2.37891	59.2
1.28	2.19122	7.336893	1.969555	5.08087	1.476247	−2.25602	44.4
3.88	1.86983	12.26883	2.369351	10.08988	2.421863	−2.17895	21.6
7.56	1.73913	8.931182	3.697961	6.627128	2.713853	−2.30405	34.8
3.56	1.68759	19.68856	15.695	15.62201	4.22624	−4.06655	26.0
3.72	1.64661	23.5168	6.926321	26.75966	7.677015	3.242852	−12.1
2.72	1.59706	7.308311	0.809606	8.84356	2.070914	1.535249	−17.4
7.84	1.56286	9.005062	4.298748	6.874986	2.494041	−2.13008	30.9
0.88	1.56004	5.225581	1.31502	3.971861	0.842471	−1.25372	31.6
3.20	1.52903	10.38416	2.419885	12.26833	3.067967	1.884173	−15.4
1.36	1.50424	5.875327	1.241649	4.617884	1.165326	−1.25744	27.2
1.52	1.47182	4.091221	1.372411	2.931726	0.679603	−1.15949	39.5
3.64	1.36743	21.24291	2.927706	19.63015	2.230882	−1.61277	8.2
8.04	1.36031	4.114875	1.798802	5.746628	3.225487	1.631754	−28.4
6.32	1.32868	2.181474	0.946195	3.196211	1.063185	1.014737	−31.7
7.12	1.31352	7.523299	1.810512	9.192125	3.744832	1.668826	−18.2
7.28	1.29916	5.603135	0.951467	4.506218	1.485876	−1.09692	24.3
3.60	1.26684	10.0813	1.418628	8.709099	2.642426	−1.3722	15.8
3.80	1.25362	20.62982	4.68134	18.93676	1.794172	−1.69305	8.9
8.00	1.22489	5.299584	2.180769	4.147602	0.891356	−1.15198	27.8
2.24	1.20716	6.914553	1.201491	5.928257	1.193245	−0.9863	16.6
5.84	1.19703	4.675383	1.565235	5.880151	2.092716	1.204768	−20.5
7.36	1.15432	14.89945	6.204194	12.83476	6.039477	−2.06468	16.1
3.96	1.14727	26.08123	4.571917	24.46342	2.922645	−1.61781	6.6
6.12	1.09125	2.506214	0.708541	3.264201	0.962606	0.757987	−23.2
8.72	1.05726	1.520031	0.43754	2.235113	1.027241	0.715082	−31.9
6.72	1.05630	1.820275	0.604988	2.612203	1.262673	0.791928	−30.3
6.36	1.05137	2.220641	0.665685	2.995081	1.179953	0.77444	−25.9
8.60	1.04735	1.592131	0.508814	2.290507	0.9637	0.698376	−30.5
5.92	1.04555	2.294618	0.941447	3.097444	1.16875	0.802826	−25.9
6.56	1.04503	2.288887	0.798836	3.082522	1.216018	0.793634	−25.7
6.44	1.03204	2.343104	0.811768	3.14314	1.286103	0.800036	−25.5
3.52	1.01803	7.737351	1.810099	6.713265	1.962173	−1.02409	15.3
2.28	1.01244	9.226281	2.920357	7.950052	3.090526	−1.27623	16.1

Table S3. VIPs, mean concentrations and differences in concentrations from ^{1}H NMR spectra of postprandial urine samples of bran *vs.* control at one hour for all chemical shifts having VIP value (for the first component) greater than 1 with 95% confidence (Jack-knifing (JK).

Chemical Shift (ppm)	VIP Component 1	Bran		Control		Difference	%Difference
		Mean	S D	Mean	S D		
1.32	3.22976	14.03238	4.202006	7.251501	3.532878	−6.78088	93.5
3.04	2.86864	125.8705	35.36431	140.6509	25.05072	14.78038	−10.5
1.56	2.82864	7.656341	1.730762	3.359924	1.474532	−4.29642	127.9
2.16	2.80171	10.41921	1.486837	6.329951	1.294214	−4.08926	64.6
2.20	2.48770	7.529601	1.17753	4.224484	1.237119	−3.30512	78.2
1.28	2.47481	8.523068	1.529586	5.049026	1.458486	−3.47404	68.8
3.28	2.13642	45.76975	31.2463	36.19298	12.36631	−9.57677	26.5
3.68	2.08969	25.92788	4.657039	21.96961	2.613812	−3.95827	18.0
3.24	2.06281	11.24925	3.377741	15.48616	5.210814	4.236908	−27.4
3.52	1.83286	8.874555	1.622894	6.551043	1.325381	−2.32351	35.5
3.88	1.69588	13.0864	2.655948	10.51419	2.078541	−2.57221	24.5
3.48	1.58521	4.57805	1.62778	2.756616	0.771308	−1.82143	66.1
7.12	1.57914	7.181518	3.319688	10.1234	4.151092	2.941877	−29.1
1.52	1.46920	4.807517	0.784111	3.174042	1.449549	−1.63347	51.5
3.60	1.44529	10.71369	1.677407	8.904177	1.517849	−1.80951	20.3
0.88	1.41586	5.807304	1.119021	4.274602	1.145499	−1.5327	35.9
1.36	1.40303	6.642524	0.846141	5.156502	1.243227	−1.48602	28.8
2.32	1.35996	4.124396	1.445991	2.725516	0.363736	−1.39888	51.3
6.12	1.33917	1.743012	1.062808	3.099028	0.915711	1.356016	−43.8
2.40	1.29894	4.226376	1.454959	2.883284	0.493987	−1.34309	46.6
6.52	1.24760	1.495537	0.799044	2.609847	0.696087	1.11431	−42.7
2.08	1.23366	7.955319	1.700262	6.469628	1.358598	−1.48569	22.9
6.04	1.19758	2.352536	1.502606	3.718709	1.2259	1.366173	−36.7
3.92	1.17109	18.50879	10.04208	15.17661	8.72161	−3.33218	21.9
8.36	1.14194	1.499548	0.588641	2.481909	0.800473	0.982361	−39.6
6.48	1.13920	1.469445	0.684679	2.361819	0.423019	0.892374	−37.8
8.08	1.13438	1.495018	0.634877	2.435821	0.663232	0.940803	−38.6
8.24	1.12107	1.528618	0.815723	2.607875	1.033628	1.079257	−41.4
8.44	1.11464	1.341518	0.829322	2.313624	0.660986	0.972105	−42.0
5.92	1.10277	1.801126	0.931051	2.838936	0.864324	1.03781	−36.6
6.00	1.10246	1.740932	0.977829	2.822414	0.9943	1.081482	−38.3
3.56	1.09193	19.41944	9.454625	16.69236	4.407347	−2.72707	16.3
6.20	1.09136	1.958378	1.205285	3.157927	1.341406	1.199549	−37.9
3.72	1.08381	24.72037	7.960002	27.50995	7.370964	2.789585	−10.1
7.88	1.07787	1.312408	0.730276	2.213375	0.634138	0.900967	−40.7
1.72	1.07724	4.090233	1.499458	2.916696	1.008155	−1.17354	40.2
6.56	1.07070	1.556704	0.930317	2.533062	0.754844	0.976358	−38.5
6.72	1.06493	1.401119	0.806436	2.313751	0.650196	0.912632	−39.4
6.64	1.06436	1.524363	0.777374	2.459477	0.765956	0.935114	−38.0
6.16	1.06166	1.754865	0.930114	2.761349	0.91607	1.006483	−36.4
1.68	1.04916	4.06487	1.339947	2.958195	1.020548	−1.10668	37.4
2.28	1.03384	9.272414	3.194829	7.628114	2.517126	−1.6443	21.6
6.60	1.03281	1.473845	0.80967	2.363446	0.683028	0.889601	−37.6
8.88	1.03014	1.163835	0.73215	2.050069	0.751731	0.886234	−43.2
8.52	1.02924	1.173862	0.709709	2.007899	0.580988	0.834037	−41.5
8.40	1.02075	1.41175	0.931872	2.291947	0.546338	0.880197	−38.4
8.76	1.01721	1.22601	0.750698	2.130143	0.849487	0.904133	−42.4
6.28	1.01390	1.604322	0.888807	2.541234	0.876	0.936912	−36.9
1.76	1.01269	3.556252	1.249734	2.577541	0.658803	−0.97871	37.9
5.96	1.01143	1.715038	0.890254	2.686527	1.022677	0.971489	−36.2
9.04	1.01010	1.138126	0.694567	1.967909	0.648979	0.829783	−42.2
8.04	1.01004	3.864529	2.350451	5.503831	3.426292	1.639301	−29.8
9.16	1.00815	1.209	0.783377	2.046918	0.598297	0.837918	−40.9
8.32	1.00750	1.358208	0.787128	2.266683	0.874335	0.908475	−40.1
2.12	1.00287	7.279841	1.525802	6.139425	1.288506	−1.14042	18.6

Table S4. VIPs, mean concentrations and differences in concentrations from ^{1}H NMR spectra of postprandial urine samples of bran *vs.* control at two hours for all chemical shifts having VIP value (for the first component) greater than 1 with 95% confidence (Jack-knifing (JK).

Chemical Shift (ppm)	VIP Component 1	Bran		Control		Difference	%Difference
		Mean	S D	Mean	S D		
1.32	4.99671	14.56618	3.347656	5.068215	1.417053	−1.87403	187.4
3.04	3.69807	121.4823	32.31417	140.5462	22.52962	0.135642	−13.6
2.16	3.31868	10.37552	2.252464	5.860334	1.571695	−0.77047	77.0
1.56	3.27916	8.147486	2.46779	3.741607	1.60901	−1.17754	117.8
2.20	3.27164	8.422423	1.947318	4.019585	1.22371	−1.09535	109.5
1.28	2.69190	8.623663	1.701308	5.08087	1.476247	−0.69728	69.7
3.68	2.58043	29.29499	7.445593	21.92234	4.805783	−0.33631	33.6
3.28	2.24345	36.65182	15.80437	30.98786	6.875153	−0.18278	18.3
3.88	2.20662	14.18108	2.052555	10.08988	2.421863	−0.40548	40.5
3.24	2.13798	11.38725	4.252732	15.48453	5.350281	0.264605	−26.5
3.92	2.10417	16.14767	6.609428	11.68593	4.777831	−0.38181	38.2
1.52	2.02322	4.72478	1.480755	2.931726	0.679603	−0.6116	61.2
3.56	1.94370	21.46209	10.54476	15.62201	4.22624	−0.37384	37.4
1.24	1.89483	8.553053	5.04785	9.375028	2.237279	0.087677	−8.8
1.36	1.48712	5.867329	1.252633	4.617884	1.165326	−0.27057	27.1
0.88	1.43091	5.089939	1.164659	3.971861	0.842471	−0.2815	28.2
6.32	1.36127	1.884843	0.753703	3.196211	1.063185	0.410288	−41.0
3.80	1.35580	22.12046	4.073146	18.93676	1.794172	−0.16812	16.8
6.44	1.31358	1.859738	0.848478	3.14314	1.286103	0.408319	−40.8
3.60	1.27876	11.1747	2.525064	8.709099	2.642426	−0.28311	28.3
3.52	1.21077	8.992438	1.828222	6.713265	1.962173	−0.3395	33.9
6.12	1.16552	2.135908	0.932849	3.264201	0.962606	0.345657	−34.6
8.04	1.15442	4.053297	2.156819	5.746628	3.225487	0.294665	−29.5
2.64	1.13131	13.44189	3.893371	15.5738	5.368122	0.136891	−13.7
7.84	1.02218	5.449178	1.936268	6.874986	2.494041	0.207391	−20.7
3.40	1.01008	5.408643	2.069119	4.36044	1.906471	−0.24039	24.0
